# The Effects of Unstable Strength Training on Lower Limb Stability in Adolescent Volleyball Players in China

**DOI:** 10.3390/life16061036

**Published:** 2026-06-22

**Authors:** Kaiyuan Dong, Borhannudin Bin Abdullah

**Affiliations:** Department of Sports Studies, Faculty of Educational Studies, Universiti Putra Malaysia, Serdang 43400, Malaysia; borhannudin@upm.edu.my

**Keywords:** unstable strength training, balance, volleyball

## Abstract

Background: This study aimed to compare the effects of unstable strength training (UST) and traditional strength training (TST) on lower-body stability in adolescent volleyball players in China. Methods: This stratified randomized controlled trial recruited 62 eligible athletes from Shandong Province. Participants were assigned to either the UST group or the TST group, and both groups completed a 10-week training program. Results: Multivariate analysis of variance (MANOVA) for within-group effects revealed statistically significant improvements in all dependent variables for both the UST and TST groups (*p* < 0.05), FMS [F = 35.112, *p* < 0.001, η^2^ = 0.377]; and balance ability differences, left-side score (LS) [F = 8.268, *p* < 0.001, η^2^ = 0.125], right-side score (RS) [F = 8.094, *p* < 0.001, η^2^ = 0.122]. Furthermore, after controlling for covariates, MANCOVA analysis for between-group effects still showed statistically significant differences between the UST and TST groups on all post-test dependent variables. These differences included: functional differences, FMS [F = 34.412, *p* < 0.001, η^2^ = 0.389]; LS [F = 8.079, *p* < 0.01, η^2^ = 0.130]; and RS [F = 8.532, *p* < 0.001, η^2^ = 0.136]. Conclusion: UST is more effective than TST in improving athletes’ lower-body stability performance. Future studies should explore the application of UST in other sports and examine its effects on parameters beyond functional movement and balance.

## 1. Introduction

Volleyball is a highly competitive and technically demanding ball sport. The sport requires seamless collaboration among multiple players on the court to execute offensive and defensive transitions [1], placing extremely high demands on athletes’ physical fitness, technical skills, tactical awareness, and mental resilience. Professional volleyball players often need to perform technical movements such as spiking, blocking, jump serves, jump receptions, and digs while in mid-air [2]. This necessitates that athletes maintain high levels of physical fitness, including functional ability, balance, and muscular strength. Previous research has indicated that poor functional movement patterns and reduced stability and balance increase the risk of lower-limb injuries in volleyball players, which in turn leads to a decline in athletic performance. Therefore, improving functional ability and neuromuscular control has become one of the key objectives in modern volleyball physical training [3]. Because FMS provides a comprehensive assessment of an athlete’s motor skills from a functional movement perspective, it is considered an essential tool for the functional evaluation of volleyball players [4].

Compared to traditional strength training, the key distinction between unstable strength training and traditional strength training lies in the contact surface: traditional training uses a stable surface, whereas unstable strength training employs an unstable surface. In an early study, Behm [5] systematically defined precise methods and operational procedures for constructing an unstable training platform using tools such as balance bags, Swiss balls, BOSU balls, and balance boards, thereby achieving the goals of unstable strength training. Previous studies have demonstrated that the BOSU ball can be effectively applied to squat strength training, enabling lower-limb strength exercises in an unstable state [6,7]. However, most studies have adopted a conservative approach to the use of the BOSU ball when designing non-stable strength training programs, consistently using it in the upright position; no studies have explored the use of the BOSU ball in an inverted position.

In contrast to weight-bearing strength training, instability-based strength training interventions affect maximum muscle output due to the unstable conditions [8], and this type of training places greater emphasis on the coordination of deep muscles [9]. Furthermore, core stability and dynamic balance in volleyball are crucial for enhancing performance and preventing injuries, whereas strength training in a traditional stable environment has limited effectiveness in developing these abilities. Recent research further indicates that instability training is more effective than isolated traditional strength training in enhancing sport-specific abilities in volleyball [10], and that resistance training based on instability significantly enhances static and dynamic balance, often surpassing the effects of traditional strength training [11]. This suggests that instability-based strength training offers greater advantages than traditional strength training in improving athletes’ functional and neuromuscular control of body stability. Furthermore, regarding age, adolescence is a critical stage for neural and muscular adaptation and development, during which athletes are particularly responsive to interventions targeting technical movement control and trunk stability [12,13], making this an area of significant research interest.

Unstable strength training addresses the limitations of traditional strength training. It plays a crucial role in enhancing volleyball players’ core stability, balance, movement economy, and injury prevention capabilities, and has become an indispensable component of modern volleyball physical training [3,14,15]. However, the existing literature still has many limitations. Previous studies have focused on general functional training or core strength training [7,16], yet systematic training interventions under unstable conditions are scarce, and direct comparisons between UST and TST remain very limited. Furthermore, previous studies have employed overly simplistic assessments of functionality and stability; although grading systems were applied, evaluations were limited to the combination of the Functional Movement Screen (FMS) and the Y-Balance Test (YBT) [17,18]. Finally, participants in stability studies have predominantly been college students and older adults [19], and evidence regarding the impact on adolescent volleyball players remains relatively scarce.

In summary, although previous systematic reviews and related studies have highlighted the benefits of instability training, most of the included studies have focused on adults or mixed-sport populations, and few have employed a controlled intervention design to directly compare the effects of UST versus TST in adolescent volleyball players. This study was conducted to address these gaps through corresponding experiments and investigations.

## 2. Materials and Methods

### 2.1. Research Design

**Figure 1:**
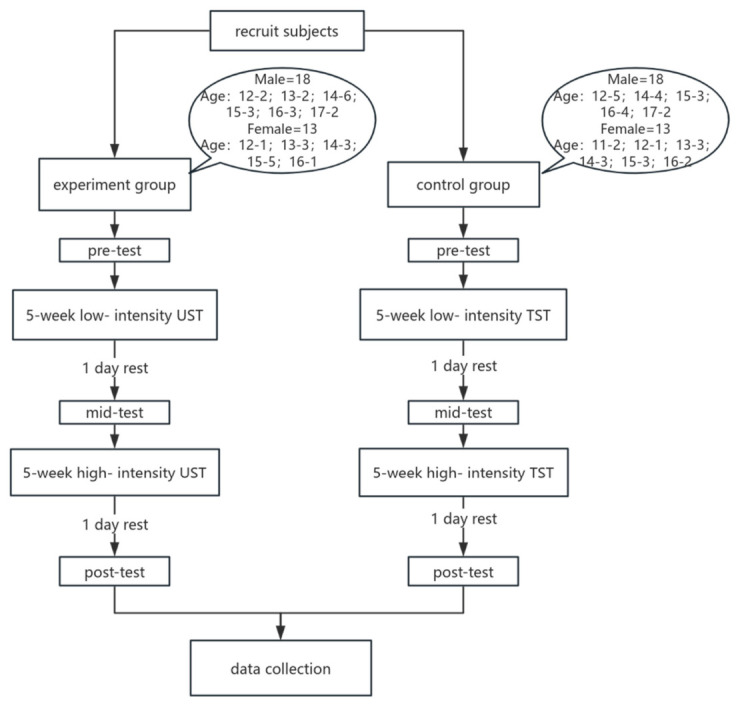
Research framework.

Participants were divided into two performance levels. To ensure that there were no significant overall differences in athletic ability between the experimental and control groups, a stratified random sampling method was employed to maintain homogeneity. Athletes were randomly selected from different level and gender before forming the experimental and control groups [20]. Specifically, first, the participants were stratified by exercise level (Level 1, Level 2); then, within each exercise level, they were further stratified by gender (male, female); finally, participants were randomly selected from each stratum to form the experimental group (Male = 18; Female = 13) and control group (Male = 18; Female = 13). This study adopted this framework for stratified sampling and implemented a training program consisting of three sessions per week over a 10-week period, based on relevant training studies [21,22]. Participants were tested before, during, and after the 10-week training intervention to collect data required for subsequent statistical analysis. This 10-week unstable strength training (UST) program was divided into two phases: a 5-week low-intensity UST phase, followed by a 5-week high-intensity UST phase. The first five weeks consisted of low-intensity functional training aimed at consolidating technical proficiency and laying the foundation for the subsequent high-intensity UST phase. This design minimized experimental errors caused by technical instability while ensuring that athletes possessed the necessary capabilities to complete the high-intensity UST program. The specific workflow is detailed in Figure 1.

### 2.2. Target Population

All volleyball players (ages 12–17) at the Shandong Provincial Sports School (Liaocheng No. 1 Experimental Sports School, Shandong Province).

### 2.3. Sample Size Calculation

**Figure 2:**
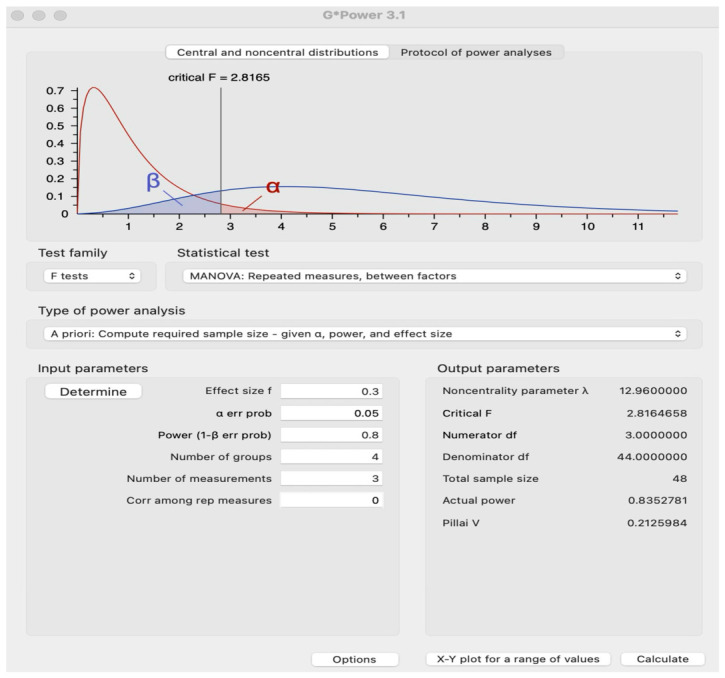
Sample size calculation using G*Power 3.1 software.

This study used G*Power 3.1 software to calculate the sample size. Based on existing literature in the field of sports training, an effect size of 0.3 was determined. Previous research on the effects of resistance training on vertical jump height in female volleyball players reported an effect size of 0.38. Another study examining the effects of strength training on the basic physical fitness of adolescent volleyball players also cited an effect size of 0.34. Based on this, we conservatively selected an effect size of 0.30 (lower than 0.34) as the expected value for sample size calculation. With a significance level of 0.05 and a power of 0.80—settings consistent with the calculation benchmarks used in previous studies evaluating athlete performance—the final sample size required for a simple effect size was determined to be 48 (Figure 2).

After accounting for a 20% dropout rate, the final minimum sample size determined for this experiment was 58 participants. Following screening, 62 subjects met the inclusion criteria and were eligible to participate in the experiment.

### 2.4. Sampling Criteria

Inclusion Criteria: 1. At least two years of training experience and a sports proficiency level of Grade 2 or higher. 2. No sports-related injuries in the past month. Athletes’ admission records clearly document their years of training and technical proficiency, facilitating direct assessment. 3. Athletes must be in good health, and their admission records must fully document any congenital conditions. During daily training, coaches also record athletes’ injuries and conduct corresponding verifications to ensure compliance with relevant standards. 4. Guardians must fully understand the experiment’s content and sign an informed consent form. A group meeting for parents will be held prior to the experiment to explain the specific details and objectives in detail, and to obtain signed informed consent forms from guardians.

Exclusion Criteria: 1. Athletes with less than two years of sports experience and a competitive level below Grade 2. During the review of athlete records, ineligible candidates will be eliminated, retaining only those who meet the criteria for training duration and competitive level. 2. Athletes with a history of prior injuries that could affect the performance of relevant technical movements. Athletes with sports injuries that impair the execution of relevant technical movements should be excluded. For example, if pain occurs during an FMS test movement, a score of 0 will be recorded and the subsequent test will be terminated. 3. Athletes with congenital heart disease. Such athletes may experience sudden cardiac events during high-intensity training interventions, posing a life-threatening risk. 4. Athletes whose guardians do not support or cooperate with this study. If a guardian refuses to cooperate, it may lead to potential risks. To ensure physical and mental health and safety, such athletes will be excluded.

### 2.5. Intervention Protocol

The control group underwent traditional strength training (TST) as shown in Table 1, with low-intensity training from weeks 1–5 and high-intensity training from weeks 6–10. The experimental group underwent unstable strength training (UST) as shown in Table 2, with low-intensity training from weeks 1–5 and high-intensity training from weeks 6–10. The training frequency was three times per week, as detailed in Table 3. Apart from differences in technical movements due to the use of unstable equipment such as BOSU balls and balance bags, all other aspects of the training protocols—including load and frequency—were identical for both groups.

During the intervention period, all participants were subject to a standardized daily schedule managed by assistant coaches. All daily meals were served in the cafeteria on the closed campus and distributed according to a meal-rationing system based on the number of participants. Participants were subject to closed-campus management during the intervention period and were not permitted to leave the premises. Due to the adoption of a professional sports team management system, the participants’ attendance rate was 100%.

### 2.6. Measurement and Instruments

First, regarding the collection of physiological data, height and weight measurements were taken using an electronic height and weight scale (OEM). Subsequently, each participant recorded their own height and weight, entered their age, and signed the form. Predicted adult height (PAH) as maturation height (MH) was estimated according to the Khamis–Roche method [23] using participants’ actual age, height, weight and their parents’ height.

Second, the FMS test is used to assess an individual’s physical movement function through specialized testing tools. Each test is administered by an assistant coach who has received standardized training and is proficient in operating the testing equipment. The total score from the seven test movements constitutes the subject’s FMS test result.

Finally, the Y-Balance Test scores effectively reflect the subject’s lower-body balance ability. The YBT kit was used to measure the subject’s leg length and scores in three directions: forward, left rear, and right rear. The data was processed using Excel, and the results were recorded for subsequent analysis.

### 2.7. Preliminary Studies

A two-week pilot study was conducted according to the experimental design to verify the feasibility of the experiment and calibrate the testing tools. Existing pilot studies have indicated that the sample size for such studies can be very small. Other studies in the field of exercise training have also noted that the sample size for pilot studies typically ranges from 6 to 15 participants [24]. This study randomly assigned 8 participants to an experimental group and a control group for a two-week pilot study, with data assessments conducted before and after the experiment. The measurement indicators required for the experiment (height, weight, and the functional movement score and YBT) were measured twice: once before the experiment began and again 48 h later. As shown in Table 4, the extremely high ICC values (ICC > 0.9) indicate that the FMS and YBT scores demonstrate a high degree of standardization and reliability across different tests, thereby validating the feasibility of the testing methods and the accuracy of the equipment. This ensured the reliability of the test data required for the experiment, allowing subsequent data analysis to draw precise conclusions and avoiding discrepancies in conclusions caused by biases in physiological data collection. After confirming accuracy, the standard experimental intervention protocol was implemented.

## 3. Result

### 3.1. Hypothesis Testing

Before conducting statistical analyses such as multivariate analysis of variance (MANOVA) and multivariate analysis of covariance (MANCOVA), it is necessary to verify the normality of the data. This study employed two commonly used statistical measures to assess normality: skewness and kurtosis. Kim and Mishra noted that data conform to a normal distribution when skewness values range from +3 to −3, kurtosis values range from +7 to −7, and the *p*-value is <0.05. This study used skewness and kurtosis to assess the normality of demographic variables and dependent variables. The results show that the skewness values for both sets of variables fall within the range of (+3 to −3), and the kurtosis values fall within the range of (+7 to −7), exhibiting typical characteristics of a normal distribution. Therefore, the demographic and anthropometric variables in both the UST and TST groups satisfy the assumption of normal distribution. The pre-test and post-test data for the dependent variables also do not violate the assumption of normal distribution. See Table 5 and Table 6 for details.

### 3.2. Descriptive Statistical Analysis

The baseline values for demographic and anthropometric characteristics are shown in Table 7. After conducting independent samples *t*-tests on each variable, the *p*-values were all greater than 0.05, indicating that there were no significant differences between the experimental group and the control group at any baseline level.

#### 3.2.1. Descriptive Statistics for FMS

Table 8 shows that the scores on the three FMS tests for female volleyball players in both the experimental and control groups were higher than those of male athletes. The mid-test and post-test scores for the experimental group were higher than those of the control group, and the degree of improvement in the experimental group was also greater than that in the control group.

#### 3.2.2. Descriptive Statistics for the YBT

Table 9 and Table 10 show that both the pre-test and post-test scores in the experimental group were higher than those in the control group, and the degree of improvement was also greater than that of the control group. The degree of improvement in the right-side scores was greater than that in the left-side scores, which is related to the dominant leg characteristic in volleyball.

### 3.3. Multivariate Statistical Analysis

MANOVA revealed significant between-group effects for group (Wilks’ Λ, F = 5.514, *p* = 0.002, η^2^ = 0.228) and gender (F = 5.465, *p* = 0.002, η^2^ = 0.226), whereas the group × gender interaction was not significant (F = 1.079, *p* = 0.365, η^2^ = 0.055) (Table 11).

Significant within-subject effects were observed for time (F = 27.302, *p* < 0.001, η^2^ = 0.756) and the time × group interaction (F = 8.150, *p* < 0.001, η^2^ = 0.480). However, the time × gender interaction was not statistically significant (F = 2.107, *p* = 0.068, η^2^ = 0.193). A significant time × group × gender interaction effect was identified (F = 4.267, *p* = 0.001, η^2^ = 0.326).

For Table 12, further analysis demonstrated significant time effects for FMS (F = 85.559, *p* < 0.001, η^2^ = 0.596), LS (F = 8.295, *p* = 0.003, η^2^ = 0.125), and RS (F = 33.726, *p* < 0.001, η^2^ = 0.368). Significant time × group interactions (F = 35.112, *p* < 0.001, η^2^ = 0.377), LS (F = 8.268, *p* < 0.001, η^2^ = 0.125), and RS (F = 8.094, *p* < 0.001, η^2^ = 0.122) were also observed for FMS, LS, and RS, indicating different improvement trajectories between the UST and TST groups across the intervention period.

**Figure 3:**
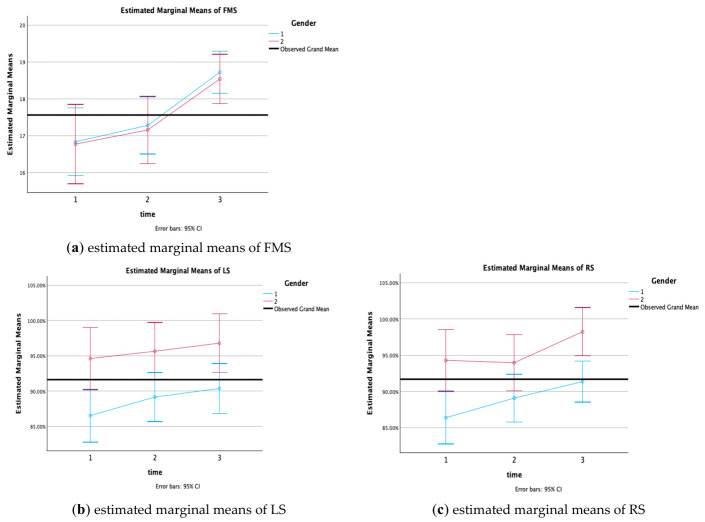
Graph of estimated marginal means.

Regarding performance by gender, the graph of estimated marginal means for the experimental group (Figure 3) shows that male and female volleyball players exhibited nearly identical trends of improvement in their FMS and LSs across the three test sessions, with all test scores showing corresponding increases. This finding is consistent with the results of the multivariate analysis. Female athletes in the RS group exhibited a fluctuating upward trend in their scores, which differed slightly from the stable upward trend observed in male athletes; however, there was still a significant difference in improvement between the post-test and pre-test scores, which is also consistent with the results of the multivariate analysis.

### 3.4. Multivariate Statistical Analysis Controlling for Covariates

After conducting a multicollinearity test on the covariates age, gender, weight, and predicted height against the FMS and YBT scores, which confirmed the absence of strong multicollinearity, a MANCOVA analysis was performed.

As Table 13 and Table 14, after controlling for age, gender, weight, and maturation height, MANCOVA results remained significant for both group effects (F = 5.582, *p* < 0.01, η^2^ = 0.237) and time × group interactions (F = 8.687, *p* < 0.001, η^2^ = 0.505). Further analysis demonstrated significant time × group effects for FMS (F = 38.785, *p* < 0.001, η^2^ = 0.409), LS (F = 10.671, *p* = 0.002, η^2^ = 0.160), and RS (F = 15.439, *p* < 0.001, η^2^ = 0.217), suggesting that the intervention effects remained robust after adjustment for covariates.

## 4. Conclusions and Discussion

### 4.1. Conclusions

UST had a positive effect on the participants’ FMS and YBT scores, and under equivalent loads, UST resulted in greater improvements in FMS and TBT for adolescent volleyball players than TST. Although there was a slight difference in the rate of improvement in balance between female and male athletes, the intervention remained effective for both genders and had a beneficial effect. Furthermore, the intervention remained significant even after controlling for age, gender, weight, and maturation height.

### 4.2. Discussion

This study examined the effects of unstable strength training (UST) and traditional strength training (TST) on the Functional Movement Screen (FMS) and Y-Balance Test (YBT) performance of adolescent volleyball players. Both interventions improved performance on the FMS and YBT; however, at the conclusion of the 10-week intervention period, the UST group demonstrated significantly greater improvements. These findings suggest that UST may be superior to TST in enhancing neuromuscular control; however, further analysis involving electromyographic testing is needed to determine the specific effects.

The significant improvement in FMS performance observed in the UST group may be attributed to the increased neuromuscular and proprioceptive demands imposed by the unstable conditions. Athletes must continuously adjust their body posture and muscle coordination while performing technical movements. These increased sensorimotor demands may enhance intermuscular coordination and trunk stability, ultimately contributing to the development of more efficient movement patterns [25]. Previous electromyography studies have also reported higher activation of stabilizing muscle groups during resistance training on unstable surfaces compared to training on stable surfaces [6,26].

Furthermore, research indicates that instability training can improve movement efficiency by enhancing proprioceptive feedback and promoting synergistic contraction strategies around major joints [8]. These adaptive changes may partially explain the significant improvements in functional movement quality observed in this study. The purpose of this study was to investigate the impact of the intervention on overall FMS performance; therefore, only the total scores were analyzed. In future research, to examine the intervention’s effects on the seven specific FMS exercises in greater detail, the test scores for each of the seven exercises could be analyzed separately, thereby allowing for a more thorough exploration of the intervention mechanisms.

The findings regarding dynamic balance performance are also consistent with previous literature on unstable surface training. Volleyball techniques require athletes to transition between various movement states, such as the spiking position in the air, the blocking position in the air, and the defensive stance on the ground; all of these movements demand a high level of dynamic muscle control and lower-body stability. The greater improvement in YBT performance observed in the UST group suggests that instability-based training may provide more sport-specific neuromuscular stimulation than traditional resistance training. Previous studies have shown that instability resistance training enhances postural control by increasing the activation of stabilizing muscles in the ankle, hip, and trunk, while simultaneously improving sensorimotor integration [10,27]. Therefore, improved neuromuscular control and enhanced physical functionality may contribute to better balance performance during dynamic movement tasks. The purpose of this study was to investigate the effects of the intervention on lower limb balance performance; therefore, only the left and right lower limb scores were analyzed. In future research, to further explore the effects of the intervention on differences in left–right balance, the left–right difference scores could be analyzed alongside the left–right balance scores, thereby providing a more comprehensive understanding of the intervention’s effects.

Another important finding was that, although there were slight differences in the rate of improvement in balance between male and female athletes, both groups demonstrated enhanced balance ability. Previous studies have noted gender differences in postural control strategies and neuromuscular activation patterns during exercise [28]. The observed differences may be associated with sex-related variations in neuromuscular control strategies; however, the underlying mechanisms remain unclear and warrant further investigation. Nevertheless, no significant gender interactions were found in most outcome variables, indicating that UST can be effectively applied to both male and female adolescent volleyball players.

The findings of this study are of particular relevance to adolescent athletes, as the neuromuscular system is highly adaptable to stimulation during adolescence. Previous studies have shown that improving movement quality and balance during adolescence may reduce the risk of injury and enhance long-term athletic performance [12,29]. Poor movement patterns and lower-body asymmetry have been linked to an increased risk of non-contact injuries in volleyball players. Therefore, incorporating instability strength training into physical training programs for adolescent volleyball players may offer benefits in both enhancing athletic performance and preventing injuries.

## 5. Limitations

This study has several limitations. First, the sample was limited to adolescent volleyball players at a sports school in Shandong Province, China; the use of a single school may limit the generalizability of the findings, and future studies could expand the recruitment scope of participants. Second, the intervention period lasted only 10 weeks, failing to examine long-term adaptive changes; furthermore, no delayed post-test was conducted after the intervention, preventing an investigation of the effects some time after the intervention ended. Future studies could appropriately increase the duration of the intervention and include delayed post-tests. Third, this study did not incorporate biomechanical or electromyography (EMG) data, which to some extent limited the interpretation of neuromuscular adaptation mechanisms. Future studies should broaden their scope to include force analysis and neuromuscular activation assessments to further elucidate the mechanisms underlying unstable strength training interventions.

Finally, although the training environment, nutritional intake, sleep schedules, and living conditions were controlled during the intervention, factors such as the off-training environment, the level of cooperation between participants and trainers, and psychological changes in participants during the intervention period may all influence the test results to some extent. This is also one of the limitations of this study. Future research could address these limitations by implementing appropriate controls.

## Figures and Tables

**Table 1:** TST program.

Content	Time	Rest
Warm-up exercises: Jogging, stretching; warm-up routine	10–15 min	
Core Muscle Activation	10 min	
Rope Ladder Coordination Training	10 min	
Alternate jumping over low hurdles with left and right feet	L*20*3, R*20*3	10 s
Glute and Hamstring Activation: Static Squat, Squat Step-Through, Static Squat Skating	10 min	
Weighted Barbell Squat	45 kg*15, 50 kg*14, 60 kg*13, 65 kg*12, 70 kg*11, 75 kg*10, 80 kg*9, 85 kg*8	1 min
Deadlift	20 kg*16*2, 30 kg*14*2, 40 kg*12*2, 50 kg*10*2	20–30 s
Power Clean	35 kg*8*2, 40 kg*7*2, 45 kg*6*2	20–30 s
Single-Leg Resistance Band Lunge	L*16*3, R*16*3	10 s
Push-ups	20*4	15–20 s
Single-Leg Half Squat	L*20*3, R*20*3	10 s
Bench press	30 kg*16, 35 kg*14, 40 kg*12, 45 kg*10, 50 kg*8, 55 kg*6	20–30 s
One-Legged Left–Right Throw and Catch of a Solid Ball	8 kg*L*16*3, 8 kg*R*16*3	10 s
Russian Spinning Body	5 kg*30*2, 10 kg*30*2	20–30 s
Wall-supported arch	20 kg*20*2, 30 kg*17*2, 40 kg*15*2	20–30 s
Relaxation and Stretching Release	15–20 min	

Note: L = left side; R = right side; *: multiplication sign.

**Table 2:** UST program.

Content	Time	Rest
Warm-up exercises: Jogging, stretching; warm-up routine	10–15 min	
Core Muscle Activation	10 min	
Rope Ladder Coordination Training	10 min	
Alternate jumping over low hurdles with left and right feet	L*20*3, R*20*3	10 s
Glute and Hamstring Activation: Static Squat, Squat Step-Through, Static Squat Skating	10 min	
Inverted BOSU Ball Weighted Barbell Squat	45 kg*15, 50 kg*14, 60 kg*13, 65 kg*12, 70 kg*11, 75 kg*10, 80 kg*9, 85 kg*8	1 min
Single-Leg Deadlift	20 kg*18*1*L&R, 30 kg*16*1*L&R, 40 kg*14*1*L&R, 50 kg*12*1*L&R	20–30 s
Power Clean	35 kg*8*2, 40 kg*7*2, 45 kg*6*2	20–30 s
Single-Leg Balance Capsule Resistance Band Leg Openers	L*16*3, R*16*3	10 s
Inverted BOSU Ball Push-Up	20*4	15–20 s
Single-Leg Balance Capsule Half Squat	L*16*3, R*16*3	10 s
BOSU Ball Bench Press	30 kg*16, 35 kg*14, 40 kg*12, 45 kg*10, 50 kg*8, 55 kg*6	20–30 s
One-Legged BOSU Ball: Throwing and Catching a Solid Ball Left and Right	5 kg*L*16*3, 5 kg*R*16*3	10 s
BOSU Ball Russian Spinner	5 kg*30*2, 10 kg*30*2	20–30 s
Wall-supported arch	20 kg*20*2, 30 kg*17*2, 40 kg*15*2	20–30 s
Relaxation and Stretching Release	15–20 min	

Note: L = left side; R = right side. *: multiplication sign.

**Table 3:** Training schedule.

	Monday	Tuesday	Wednesday	Thursday	Friday	Saturday	Sunday
8:30 a.m.–12:00 a.m.	Skills	Cultural Classes	Skills	Cultural Class	Skills	Skills Summary	Rest
Lunch Break							
2:00 p.m.–5:30 p.m.	Counterattacks	UST/TST	Skills	UST/TST	Skills	UST/TST	Rest

Note: UST= Unstable Strength Training; TST = Traditional Strength Training.

**Table 4:** Test–retest reliability of testing instruments.

Variable	Measuring Method	(ICC)	95% Confidence Interval	
			Lower Bound	Upper Bound
Functional Ability	FMS	0.992	0.986	0.995
Balance Ability	YBT-LS	0.998	0.996	0.999
	YBT-RS	0.994	0.990	0.996

Note: FMS = Functional Movement Screen; YBT = Y-Balance Test; LS = Left Score; RS = Right Score; ICC = Intraclass Correlation Coefficients.

**Table 5:** Normality test of demographic and anthropometric characteristics variables.

Variables	Minimum	Maximum	Mean	SD	Skewness	Kurtosis
Age	11	17	14.27	1.506	−0.100	−0.726
Weight (kg)	42	95	63.37	9.105	0.467	1.424
MH (cm)	163	206	182.69	8.971	−0.141	−0.122

Note: MH = maturation height.

**Table 6:** Normality test of pre/post-test of dependent variables for UST and TST groups.

Variables	UST Group (N = 31)						TST Group (N = 31)					
	Skewness			Kurtosis			Skewness			Kurtosis		
	**Pre**	**Post 1**	**Post 2**	**Pre**	**Post 1**	**Post 2**	**Pre**	**Post 1**	**Post 2**	**Pre**	**Post 1**	**Post 2**
FMS	−1.26	−0.52	1.17	−0.71	−0.56	−0.78	−1.38	−1.24	−1.12	−4.90	−4.65	−4.32
Left Score	0.49	0.42	0.49	−1.48	−0.98	−1.05	−0.42	−0.42	−0.42	−1.21	−1.21	−1.21
Right Score	0.49	0.42	0.49	−1.48	−0.98	−1.05	−0.12	−0.12	−0.12	−0.82	−0.82	−0.82

Note: UST = unstable strength training, TST = traditional strength training.

**Table 7:** The baseline on demographic and anthropometric characteristic for UST and TST groups.

Variables	UST Group (N = 31)	TST Group (N = 31)
Age (years)	14.35 ± 1.36	14.69 ± 1.18
Height (cm)	176.29 ± 8.63	175.07 ± 8.77
Maturation Height (cm)	181.35 ± 8.77	179.86 ± 9.04
Weight (kg)	63.77 ± 10.13	61.63 ± 10.79

Note: UST = unstable strength training; TST = traditional strength training.

**Table 8:** FMS descriptive statistics table.

Variable	Group	Mean	Std.	Min	Max.
FMS1	1	16.81	1.87	13	20
	2	16.84	2.19	9	20
FMS2	1	17.23	1.59	14	20
	2	16.90	2.17	9	20
FMS3	1	18.65	1.17	16	20
	2	17.26	2.00	10	20

Note: group 1 = experiment group, group 2 = control group.

**Table 9:** YBT-LS descriptive statistics table.

Variable	Group	Mean	Std.	Min	Max.
LS1	1	89.91%	8.70%	73.88%	111.37%
	2	87.11%	6.39%	71.79%	98.08%
LS2	1	91.87%	7.81%	75.76%	111.04%
	2	86.41%	5.77%	71.79%	95.71%
LS3	1	93.06%	7.87%	75.70%	112.25%
	2	87.07%	7.16%	70.94%	102.87%

Note: group 1 = experiment group, group 2 = control group.

**Table 10:** YBT-RS descriptive statistics.

Variable	Group	Mean	Std.	Min	Max.
RS1	1	89.69%	8.39%	76.53%	109.58%
	2	85.22%	6.04%	71.38%	94.60%
RS2	1	91.11%	7.13%	77.31%	108.08%
	2	84.60%	6.68%	70.97%	94.04%
RS3	1	94.24%	6.73%	82.10%	110.08%
	2	86.24%	6.45%	72.78%	100.00%

Note: group 1 = experiment group, group 2 = control group.

**Table 11:** Multivariate tests of MANOVA.

Effect	F (Wilks’ Lambda)	Eta2
between subjects		
group	5.514 **	0.228
gender	5.465 **	0.226
group × gender	1.079	0.055
within subjects		
time	27.302 ***	0.756
time × group	8.150 ***	0.48
time × gender	2.107	0.193
time × group × gender	4.267 *	0.326

Noted: * *p* < 0.05, ** *p* < 0.01, *** *p* < 0.001 means significant differences.

**Table 12:** Comparison between variables of MANOVA.

Variable	Time		Time × Group		Time × Gender		Time × Group × Gender	
	**F**	**Eta^2^**	**F**	**Eta^2^**	**F**	**Eta^2^**	**F**	**Eta^2^**
FMS	85.559 ***	0.596	35.112 ***	0.377	0.949	0.016	0.341	0.006
LS	8.295 ***	0.125	8.268 ***	0.125	1.054	0.018	2.749	0.045
RS	33.726 ***	0.368	8.094 ***	0.122	2.651	0.044	12.584 ***	0.178

Noted: *** *p* < 0.001 means significant differences.

**Table 13:** Multivariate tests effects of MANCOVA.

Variables	F	Eta
between subjects		
Age	3.673 *	0.169
Weight	1.152	0.060
MH	0.198	0.011
Gender	1.766	0.089
Group	5.582 **	0.237
within subjects		
Time	0.912	0.097
Time × Age	1.019	0.107
Time × Weight	0.388	0.044
Time × MH	0.783	0.084
Time × Gender	1.483	0.149
Time × Group	8.687 ***	0.505

Note: MH = maturation height; *p* < 0.05 *; *p* < 0.01 **; *p* < 0.001 ***.

**Table 14:** Comparison between variables of MANCOVA.

Variable	Time		Time × Group		Time × Gender		Time × Group × Gender	
	**F**	**Eta^2^**	**F**	**Eta^2^**	**F**	**Eta^2^**	**F**	**Eta^2^**
FMS	1.008	0.018	38.785 ***	0.409	0.209	0.004	0.343	0.007
LS	1.702	0.029	10.671 **	0.160	0.867	0.015	2.689	0.042
RS	1.085	0.019	15.493 ***	0.217	3.694	0.062	11.973 ***	0.169

Noted: ** *p* < 0.01, *** *p* < 0.001 means significant differences.

## Data Availability

The data presented in this study are available on request from the corresponding author due to privacy of the participants.

## Abbreviations

The following abbreviations are used in this article:

UST	Unstable strength training
TST	Traditional strength training
FMS	Functional Movement Screen
YBT	Y-Balance Test
PAH	Predicted adult height

## References

[1] Paul D.J., Gabbett T.J., Nassis G.P. (2016). Agility in team sports: Testing, training and factors affecting performance. Sports Med..

[2] Garcia S., Delattre N., Berton E., Divrechy G., Rao G. (2022). Comparison of landing kinematics and kinetics between experienced and novice volleyball players during block and spike jumps. BMC Sports Sci. Med. Rehabil..

[3] Kızılet T. (2021). The effects of high-intensity functional training on aerobic capacity, metabolic adaptation and neuromuscular responses in young female volleyball players. Eur. J. Mol. Clin. Med..

[4] Uysal G.E., Baydemir B. (2026). Functional movement screen and asymmetries in female volleyball players across playing positions. Sci. Rep..

[5] Behm D.G., Colado Sanchez J.C. (2013). Instability resistance training across the exercise continuum. Sports Health.

[6] Behm D.G., Muehlbauer T., Kibele A., Granacher U. (2015). Effects of strength training using unstable surfaces on strength, power and balance performance across the lifespan: A systematic review and meta-analysis. Sports Med..

[7] Gao J., Liu D., Zhu J., Guo Q., Wang X. (2025). Instability core training vs traditional core training on trunk strength and sprint performance among athletes: A systematic review and meta-analysis. PeerJ.

[8] Zemková E. (2017). Instability resistance training for health and performance. J. Tradit. Complement. Med..

[9] Gündoğan B., Aydın E.M., Sağlam A.F. (2023). Muscle Activation during Squat on Different Surfaces. Pamukkale J. Sport Sci..

[10] Zemková E., Zapletalová L. (2022). The role of neuromuscular control of postural and core stability in functional movement and athlete performance. Front. Physiol..

[11] Abdullah B.B., Dev R.D.O. (2024). Effect of instability resistance training on core muscle strength among athletes: A systematic review. Int. J. Hum. Mov. Sports Sci..

[12] Zarei M., Shabani A., Mohammadi V. (2022). Effects of core stability training on balance and performance. J. Sports Sci. Med..

[13] Chomiuk T., Kasiak P., Filipek A., Mamcarz A., Śliż D. (2025). Functional Movement Screen scores are comparable in volleyball players with and without back pain—The FMS-VBP study. J. Clin. Med..

[14] Tsai Y.-J., Chia C.-C., Lee P.-Y., Lin L.-C., Kuo Y.-L. (2020). Landing kinematics, sports performance, and isokinetic strength in adolescent male volleyball athletes: Influence of core training. J. Sport Rehabil..

[15] Yapıcı A. (2019). Effects of 6 weeks core training on balance, strength and service performance in volleyball players. Eur. J. Phys. Educ. Sport Sci..

[16] Yu T., Xu Y., Zhang Z., Sun Y., Zhong J., Ding C. (2025). The impact of core training on overall athletic performance in different sports: A comprehensive meta-analysis. BMC Sports Sci. Med. Rehabil..

[17] Erol M., Gözlükaya Girginer F., Seyhan S., Acar G., Cerit G., Uzun M., Soylu C. (2025). Predicting injury risk in young female volleyball players through movement and jump assessments. Front. Public Health.

[18] Younis M.S., Ebid A.A., Al Zughaibi K.M., Abdelgalil A.A., Sannan H.M., Ibrahim A.R. (2023). Anthropometric and jumping profiles of U19 boys’ volleyball players in the World Championship 2019. J. Complement. Med. Res..

[19] Zhitao L., Junlong D., Rui Y., Leijiao Y., Cheng G., Jun Y. (2024). Relationships between functional movement quality and sports performance in elite figure skating athletes of China. J. Strength Cond. Res..

[20] Chipman J.J., Mayberry L., Greevy R.A. (2022). REMATCHING ON-THE-FLY: Sequential matched randomization and a case for covariate-adaptive randomization. arXiv.

[21] Lasevicius T., Schoenfeld B.J., Grgic J., Laurentino G., Tavares L.D., Tricoli V. (2019). Similar muscular adaptations in resistance training performed two versus three days per week. J. Hum. Kinet..

[22] Matuszczyk F., Trybulski R., Gałęziok K., Olaniszyn G., Terbalyan A., Wilk M. (2025). Effect of 10-week plyometric training on anaerobic performance and biomechanical properties of the muscles in football players: Randomized controlled trial. Appl. Sci..

[23] Khamis H.J., Roche A.F. (1994). Predicting adult stature without using skeletal age: The Khamis-Roche method. Pediatrics.

[24] Teresi J.A., Yu X., Stewart A.L., Hays R.D. (2022). Guidelines for designing and evaluating feasibility pilot studies. Med. Care.

[25] Bertozzi F., Brunetti C., Maver P., Galli M., Tarabini M. (2025). The role of age and maturation on jump performance and postural control in female adolescent volleyball players over a season. BMC Sports Sci. Med. Rehabil..

[26] Sparkes R., Behm D.G. (2010). Training adaptations associated with an 8-week instability resistance training program with recreationally active individuals. J. Strength Cond. Res..

[27] Drinkwater E.J., Pritchett E.J., Behm D.G. (2007). Effect of instability and resistance on unintentional squat-lifting kinetics. Int. J. Sports Physiol. Perform..

[28] Çakır M., Ergin E. (2022). The Effect of Core Training on Agility, Explosive Strength and Balance in Young Female Volleyball Players. J. Sport Sci. Res..

[29] Pfeifer K., Banzer W., Ferrari R. (2019). Functional movement and injury prevention. Sports Med..

